# The Short- and Long-Term Effects of a Short Course of Sauerkraut Supplementation on the Gut Microbiota of Active Athletes: A Pilot Follow-Up Study

**DOI:** 10.3390/nu17050929

**Published:** 2025-03-06

**Authors:** Jadran Zonjić, Andrija Karačić, Ivona Brodić, Antonio Starčević, Ira Renko, Željko Krznarić, Matija Ivančić, Ana-Marija Liberati Pršo, Zvonimir Šatalić

**Affiliations:** 1 Faculty of Food Technology and Biotechnology, University of Zagreb, Pierottijeva 6, 10000 Zagreb, Croatia; jadran.zonjic@gmail.com (J.Z.); andrija@ccm.hr (A.K.); ivona.nutricionist@gmail.com (I.B.); antonio.starcevic@gmail.com (A.S.); ira.renko@gmail.com (I.R.); zvonimir.satalic@pbf.unizg.hr (Z.Š.); 2 The Gut Microbiome Center (CCM), Jablanska 82, 10000 Zagreb, Croatia; 3 Department of Internal Medicine, University Hospital “Sveti Duh”, Sveti Duh 64, 10000 Zagreb, Croatia; matijai2307@gmail.com; 4 Department of Internal Medicine, Faculty of Medicine, University of Zagreb, 10000 Zagreb, Croatia; zeljko.krznaric60@gmail.com

**Keywords:** sports nutrition, sauerkraut, microbiota, personalization, fermented food, whole food, athletes

## Abstract

**Objectives**: The application of whole fermented foods in sports nutrition for gut microbiota personalization is a promising area of investigation. Sauerkraut, a traditional fermented food, has not been extensively studied in this context. This study aimed to replicate earlier findings of a proof-of-concept study on the potential of sauerkraut for gut microbiota personalization in active athletes. **Methods**: A pilot follow-up study was conducted on active recreational athletes who consumed 250 g of organic pasteurized sauerkraut daily for 10 days. Changes in gut microbiota composition and functionality were assessed using 16S rRNA sequencing and metabolic pathway analysis across three time points: pre-intervention, postintervention, and one-month postintervention. Laboratory parameters, bowel function, and side effects were monitored throughout the study. **Results**: In total, 11 male participants with an average age of 30 years completed the study. The pilot follow-up study did not replicate the original study’s findings on sauerkraut’s short-term effects on β-diversity and taxonomic and functional groups. However, long-term effects of sauerkraut supplementation were demonstrated, including a significant reduction in α-diversity variance and increased gut microbiota composition similarity (β-diversity) as well as several significant changes in bacterial taxa and metabolic pathways after a washout period. The intervention also induced a transient decrease in B12 vitamin levels and a short- and long-term increase in leukocyte concentrations. The probability for physiological types of stools increased after one week of sauerkraut supplementation as well as the incidence of gastrointestinal side effects, such as bloating, diarrhea, pain, nausea, and constipation. **Conclusions**: This study suggests that the short-term effects on the gut microbiota of sauerkraut supplementation depend on its baseline status, but it can induce long-term effects. Sauerkraut supplementation requires a seven-day adaptation period. Further research is needed to explore the mechanisms behind the short- and long-term effects of sauerkraut supplementation.

## 1. Introduction

The International Scientific Association for Probiotics and Prebiotics (ISAPP) defines fermented foods as “foods made through controlled microbial growth and enzymatic conversions of food components” [1]. Fermentation is a biochemical process in which microorganisms such as bacteria, yeasts, or molds metabolize organic compounds from the substrate—usually sugars and starches—typically under anaerobic conditions, producing various derivatives that enhance the nutritional, organoleptic, and technological properties of the food [2]. Fermented foods are an important component of a healthy human diet [3]. In recent years, there has been a growing global consensus that the consumption of fermented foods is associated with positive health effects [4].

The health effects of fermented foods have been extensively studied and are well documented [5,6]. Thanks to next-generation sequencing and metagenomic methods, it is now possible to also precisely track the effects of fermented foods on the health of the gut microbiota as well. In cross-sectional studies, the gut microbiota of individuals who regularly consume plant-based fermented foods differs significantly in terms of β-diversity and contains higher proportions of *Bacteroides* spp., *Prevotella* spp., *Dorea* spp., and *Lachnospiraceae* [7,8], whereas among consumers of dairy-based fermented foods (yogurt), *Streptococcus thermophilus* and *Bifidobacterium animalis* prevail [9]. Consequently, the gut microbiota is enriched not only with bacteria associated with fermented foods, including various lactic acid bacteria, but also with certain unrelated bacteria such as particular *Prevotella* and *Enterococcus* species [8]. A 2024 literature review identified 42 human interventional studies on specific fermented foods, of which 24 examined modulation of the gut microbiota [10]. Although the outcomes depend on the studied specific aliments, human interventional research generally shows that regular consumption of fermented foods increases α-diversity [11] and, depending on the specific product, may increase the proportion of bacterial groups associated with short-chain fatty acids (SCFA) production and immunomodulatory effects while reducing the proportion of potentially pathogenic bacteria [12,13,14,15,16]. Owing to the above-mentioned effects on the composition of the gut microbiota, the application of fermented foods has the potential to counteract dysbiosis, a disturbance of the gut microbiota. However, due to the heterogeneity of the studies conducted to date, this hypothesis cannot yet be confirmed by scientific evidence [17].

Whole fermented foods are emerging as key components of sports nutrition due to their multiple health effects but also availability and practicality [5,6]. Due to their potential application in targeted gut microbiota personalization, fermented foods are a promising management option for dysbiosis prevention and treatment [18]. The broad implications of dysbiosis on athletic performance and recovery [19] underline the importance of whole fermented foods in sports nutrition.

Sauerkraut is probably the most popular fermented whole food preparation in Europe [6]. Sauerkraut is a fermented vegetable product that is derived from the malolactic fermentation of raw fresh white cabbage (*Brassica oleracea* L. var. capitata) in a salt brine with 2–3% (*w*/*w*) sodium chloride [20]. Sauerkraut can be regarded as a synbiotic whole food, containing pro-, pre-, and postbiotics, all compounds with beneficial effects on the gut microbiota [21]. Sauerkraut is enriched with probiotics involved in the fermentation process [22], such as *Weissella* spp., *Leuconostoc mesenteroides*, *Levilactobacillus brevis*, *Lactiplantibacillus plantarum*, and *Pediococcus pentosaceus* [23], which are resistant to bile salts and low gastric pH (one even to β-hemolysis) and demonstrate antimicrobial activity [24]. Sauerkraut is rich in fiber by nature, which can act as a prebiotic, and biologically active metabolites, including SCFA and biogenic amines, produced by the controlled bacterial metabolism (activity and growth) [25]. Due to its synbiotic properties, sauerkraut supplementation could potentially be beneficial for gut microbiota personalization in athletes, thereby improving athletic performance and recovery [26]. The health benefits of sauerkraut consumption have been studied in a limited body of research, mostly on in vitro models [27]. Human clinical trials are still scarce. Based on earlier scientific findings, one can conclude that the use of sauerkraut can potentially exert beneficial effects on digestive function disorders, such as various functional disorders (e.g., IBS) [28]; enhance immune functions, including the body’s defense against infections and immune response to tumor processes (via glucosinolate); and positively influence metabolic disorders such as diabetes [29,30].These observed effects underline the potential of sauerkraut supplementation in sports nutrition.

Since a knowledge gap was found regarding the potential role of sauerkraut in sports nutrition for gut microbiota personalization, a proof-of-concept study was conducted in 2023 [31].

The original study was limited due to the small sample size, which was reflected in high false-positivity rates in the results regarding the gut microbiota. But even when adjusted for false positives, the proof-of-concept study yielded a comparably large number of statistically significant results. Consequently, a decision was made to replicate the study and conduct a pilot follow-up study to attempt to reproduce the positive outcomes of the intervention in a new sample of participants with similar characteristics but of greater quantity. An additional objective of the confirmatory study was to also assess the long-term effects of the intervention by including an additional sampling time point for stool and laboratory parameters measurements 30 days postintervention, thereby introducing a washout period (T40). With the newly obtained data, we further close the literature gap on the potential of the sauerkraut supplementation in sports nutrition for gut microbiota personalization.

## 2. Materials and Methods

### 2.1. Study Design

The study aimed to investigate the short- and long-term effects of a short course of sauerkraut supplementation on the gut microbiota of active athletes. The primary goal was to potentially replicate the effects observed in the original study within a similar cohort of active athletes [31]. The intervention itself was identical to that in the original study, lasting 10 days. In this study, its comparably short duration was based on earlier studies, where potent synbiotics altered the gut microbiota already after a short administration of 10 days [32,33], and chosen to facilitate study execution. However, it is important to note that the fermented food used in this study (sauerkraut) was produced a year later, in 2023. Outcome measures included changes in gut microbiota composition and laboratory parameters, as in the first study. Gut microbiota analyses followed the same protocol and were conducted in the same laboratory to minimize potential deviations due to technical aspects of amplicon sequencing or laboratory testing. Confounding factors such as diet, training, and sleep were monitored using identical research diaries [34]. In addition to the athlete diet index (ADI) [35], additional questionnaires were introduced to evaluate adherence to the Mediterranean diet, providing further objectivity regarding dietary intake during the intervention. As in the first study, stool characteristics were monitored using the Bristol Stool Scale (BSS), and potential side effects of sauerkraut intake were recorded.

Recruitment was conducted through collaboration with sports clubs and nutritionists specializing in athlete nutrition as well as via the researchers’ local networks. Unlike the first study, recreational athletes were included to facilitate and accelerate recruitment. The number of participants remained the same as in the first study.

As in the first study, an initial consultation was conducted at the research facility (The Gut Microbiome Center, CCM), where potential participants were provided with detailed verbal and written instructions about the study by the research team. Written informed consent forms were signed by both the participants and the researchers after all study procedures, risks, and benefits were thoroughly explained. Participants were provided with stool sampling kits and research materials, including sauerkraut. Body composition was assessed using bioelectrical impedance (Tanita MC-780, Tanita Corporation, Tokyo, Japan) at the initial consultation.

Participants were instructed to minimize deviations from their usual lifestyle during the intervention, particularly regarding diet, to allow precise monitoring of the intervention’s effects on gut microbiota. Therefore, they were asked to record food intake, supplementation, sleep duration, and physical activity for three to four days prior to and during the intervention. To simplify participation based on the experience from the first study, the pre-intervention monitoring period was shortened. Three questionnaires were used to objectively assess dietary intake: the athlete diet index (ADI), the Mediterranean Diet Adherence Screener (MEDAS) [36], and the Short Questionnaire on Mediterranean Diet Adherence and Diet Sustainability (SQM) [37].

To evaluate short- and long-term effects, stool sampling was performed by participants at home at three time points: the day before the intervention, the day after the intervention, and one month after the intervention, following the washout period.

The intervention consisted of daily supplementation with 250 g of sauerkraut. As this was a confirmatory study, the daily amount of sauerkraut remained unchanged from the first study. For logistical reasons, the amount was not adjusted for participants’ body weight. However, participants were allowed to vary the timing and combination of sauerkraut intake throughout the day, as long as they consumed 500 g every two days (equivalent to two daily doses). Participants recorded the amount and timing of sauerkraut intake in research diaries. Sauerkraut could be consumed alone or in combination with other foods (e.g., in salads or as a side dish). Any side effects, particularly gastrointestinal symptoms (e.g., constipation, diarrhea, bloating, flatulence, or abdominal pain), were recorded in the research diary throughout the study.

All procedures associated with this study adhered to the Declaration of Helsinki and were approved by the Ethics Committee of the University of Zagreb School of Medicine (reference number: 380-59-10106-23-111/36) on 27 March 2023. The study was registered at ClinicalTrials.gov under registration number NCT06087146.

### 2.2. Participants

The objective of this study was to recruit active athletes, as in the proof-of-concept study, to replicate its findings as closely as possible. However, due to recruitment challenges in the initial study, the inclusion criteria were broadened from professional athletes or those professionally engaged in sports to individuals engaged in regular physical activity, even at a recreational level. Athletes from various disciplines were included again to minimize the influence of any specific sport on the gut microbiota. While male participants were again included, female participants were explicitly excluded in this study. To further replicate the initial study, the median age of participants from the first study was used as an inclusion criterion. The minimal sample size was established based on the original study power analysis results, which utilized a paired *t*-test and found that a sample size of 10 participants is sufficient to detect a large effect with 80% power and a significance level of 0.05. Therefore, the aim was to enroll around 20 subjects for adequate follow-up and, in the case of recruitment difficulties, at least 10 active athletes.

The inclusion criteria were as follows:

1. Age approximately 30 years;
2. Male sex;
3. Regular weekly engagement in physical activity (minimum four trainings weekly);
4. Good general physical health (assessed through annual occupational health check-ups, medical commissions of the Olympic Committee, sports medicine, or primary healthcare).

The exclusion criteria were identical to those of the initial study:

1. Use of antibiotics within six months prior to or during the intervention;
2. Probiotic supplementation within six months prior to or during the intervention;
3. History of gastrointestinal surgery;
4. Chronic medication use;
5. Known allergy to fermented or raw cabbage.

Since professional athletes were no longer the focus of this research, the study’s timeline was not aligned with training or competition schedules. The “Participant Classification Framework” [38] was used to classify participants based on their physical activity levels and athletic achievements into different levels.

### 2.3. Supplementation Protocol

For the confirmatory study, sauerkraut from the same producer as in the proof-of-concept study, Eko Imanje Zrno d.o.o. (Vrbovec, Croatia), was used. This was pasteurized sauerkraut grown locally under biodynamic agricultural conditions. The sauerkraut was produced using the same process, recipe, personnel, and location, fermented in a 2% saline solution without preservatives. The only technological difference was that the cabbage was harvested and processed a year later, in 2023 [39].

Each participant received five 500 g jars of sauerkraut. The nutritional and microbiological properties were not re-tested; instead, results from the analysis of the sauerkraut produced the previous year (2022) were assumed.

### 2.4. Standardization of Physical Activity, Sleep, and Diet

To eliminate key confounding factors, participants recorded their lifestyle habits, specifically physical activity, diet, and sleep, in a virtual Excel research diary (Microsoft, Palo Alto, CA, USA) designed for the proof-of-concept study. Participants completed the research diary in two phases: seven days before the intervention and during the ten-day intervention. For physical activity, participants recorded the duration of training sessions, including start and end times as well as the type of training. Regarding diet, participants logged the time, quantity, and specific foods consumed in each meal. If exact quantities could not be measured using scales, the Capnutra Food Atlas for this specific region of Europe [40] was used. For sleep, participants recorded their bedtime and approximate wake-up time.

The collected data were analyzed by researchers, while dietary intake was analyzed by a nutritionist involved in the study. Dietary intake was quantified using the USDA database (U.S. Department of Agriculture, Agricultural Research Service, Beltsville Human Nutrition Research Center) to calculate energy (kilocalories) and macronutrient intake for the two phases: before and during the intervention. For physical activity and sleep, the average number of weekly training sessions, daily training duration, and average hours of sleep were calculated for both phases.

To further standardize dietary intake before and during the intervention, the athlete diet index (ADI) and two questionnaires on adherence to the Mediterranean diet were used: the Mediterranean Diet Adherence Screener (MEDAS) and the Short Questionnaire on Mediterranean Diet Adherence and Diet Sustainability (SQM).

The athlete diet index is a validated tool for assessing diet quality in professional and recreational athletes, providing an overall diet score and specific component scores (e.g., intake of key nutrients) [35].

To further evaluate dietary adherence objectively, additional questionnaires assessing adherence to the Mediterranean diet were included. The Mediterranean diet, along with plant-based diets, is considered optimal for gut microbiota composition and function, increasing diversity and short-chain fatty acid (SCFA) production [41]. The MEDAS questionnaire consists of 14 questions on the intake of key food groups in the Mediterranean diet, such as olive oil, legumes, and red wine [42]. Although validated in Croatia and globally [36,43,44], MEDAS assigns points for alcohol consumption, which has adverse effects on gut microbiota [45]. Therefore, the SQM questionnaire, which excludes alcohol, was also used. SQM is a reliable tool consisting of eight questions on key Mediterranean food groups and diet sustainability [46].

### 2.5. 16S rRNA NGS Analysis of Gut Microbiota

Participants collected stool samples at three time points: the day before the intervention (day 0, T0), the day after the intervention (day 11, T10), and one month after the intervention (day 30 after washout, T40). Samples were collected using cotton swabs and toilet paper at home, following provided instructions.

Procedures related to sample storage and the amplicon sequencing process were identical to those in the proof-of-concept study, where the process is described in detail [31].

### 2.6. Laboratory Analysis

Samples for laboratory parameters and gut microbiota were collected at three time points: the day before, the day after, and one month after the intervention. Laboratory parameters were selected based on statistically significant findings from the proof-of-concept study and included the following:

1. Blood count parameters: leukocytes, neutrophils, and lymphocytes;
2. Vitamins: vitamin B12 and folic acid.

Laboratory analysis was performed in the same tertiary healthcare facility as in the first study, using EDTA or citrate blood depending on the measurement, as described in the original study [31].

### 2.7. Statistical Analysis

Statistical analysis followed the same principles as the proof-of-concept study. Online forms were coded, and data were imported into SPSS (IBM Corp. Released 2020. IBM SPSS Statistics for Macintosh, Version 27.0. IBM Corp., Armonk, NY, USA) for descriptive and inferential statistical analyses.

Due to differences in study design, minor changes were made to the statistical analysis, particularly for outcome measures. Confounding factors, such as dietary intake and other lifestyle habits, were analyzed during two phases (pre- and postintervention). Mean differences in confounding factors were treated as quantitative variables and analyzed using paired *t*-tests after testing for distribution type.

Outcome measures, including gut microbiota and laboratory parameters, were assessed at three time points. Laboratory results were tested for distribution using the Shapiro–Wilk test. Depending on normality, differences across three time points were analyzed using repeated-measures ANOVA or the Friedman test. Two laboratory parameters, leukocyte concentration and vitamin B12 concentration, were assed at two time-points with the paired *t*-test.

Gut microbiota analysis used α-diversity metrics calculated based on ASVs subsampled to 10,000 reads per sample. Shannon’s index was calculated using the Qiime2 diversity function [47]. Hypotheses were tested using repeated-measures correlation (rmcorr 0.7.0 function in R) [48]. We performed Levene’s test for homogeneity of variances to assess the variance of α-diversity at the different time-points. *p*-values were adjusted for multiple hypothesis testing using the Benjamini–Hochberg correction, with significance set at *q* < 0.05 (non-FDR) [49].

Taxonomic composition and metabolic pathway differences were assessed using repeated-measures analysis, filtered to a minimum threshold of 0.1% average relative abundance. Centered log-ratio (clr) transformation with pseudocounts was applied, and visualizations were generated using ggplot2 3.5.1 in R [50].

Stool data were processed similarly to the first study. The probability of Bristol Stool Scale types 3 or 4 was analyzed using binomial tests in R with chi-squared tests, and statistically significant results were defined as *p*-values < 0.05.

## 3. Results

### 3.1. Participants

The present study faced similar recruitment difficulties as the original study, although the inclusion criteria were eased. Researchers found that interest in the intake of sauerkraut was very low in the active athlete population, and therefore, sample size did not exceed that of the original study. Participant data, including sociodemographic characteristics such as sex, age, sport type, and years of involvement in sports, are presented in Table 1.

The average age of 30 years in this study was one year higher than in the initial study, with a smaller standard deviation of 1.56 compared to 4 years in the original study. Because recreational athletes were included in this study, the average athlete classification score was lower (2.64 ± 0.98) than in the first study (3.9 ± 1.22). While Level 4 corresponds to athletes competing at the national level (average in the first study), Level 2 corresponds to athletes engaged in organized training.

### 3.2. Physical Activity and Sleep

Physical activity and sleep, along with dietary intake as a confounding factor, were monitored before and during the intervention. Data on average training frequency, duration, and sleep are shown in Table 2. No statistically significant differences in physical activity or sleep were observed before and during the intervention. However, average sleep duration slightly increased (*p* = 0.263), while the number of training sessions (*p* = 0.472) and their duration (*p* = 0.559) decreased.

### 3.3. Diet

Complete dietary intake was tracked before and during the intervention using virtual dietary diaries. Additional interviews with nutritionists were conducted postintervention to fill in missing details, and nutritionists constructed dietary records based on the participants’ data. Table 3 shows average daily dietary intake before and during the intervention.

Significant increases in daily fiber intake and fiber intake per 1000 kcal were observed during the intervention, likely attributed to sauerkraut consumption. Other changes in dietary intake were not statistically significant.

While the Athlete Diet Index (ADI) score was slightly lower during the intervention, the difference was not statistically significant (*p* = 1.000). Higher adherence to the Mediterranean diet was observed based on MEDAS and SQM questionnaire results, likely due to increased vegetable intake from sauerkraut. However, this change was also not statistically significant (*p* ≥ 0.0.073), as showed in Table 4.

Results from multiple independent tools suggest no significant dietary changes occurred during the intervention. Therefore, diet can likely be excluded as a potential confounding factor in interpreting the intervention’s outcomes.

### 3.4. Digestion and Side Effects

Participants recorded stool changes and any potential adverse effects of the intervention in their research diaries. Results are shown in Table 5. Statistical analysis revealed that the probability of Bristol stool types (BST) 3 and 4 increased after seven days of the intervention (HR 0.45 to 0.73). However, this increase was not statistically significant (*p* = 0.227). Throughout the first week, three participants consistently reported BST types outside the range of 3 and 4.

Reported adverse effects included bloating, diarrhea, pain, nausea, and constipation, with the highest incidence on days 5 and 7 of the intervention. Bloating was the most frequently reported side effect, followed by diarrhea.

### 3.5. Changes in Gut Microbiota

Due to the inadequacy of one participant’s stool samples at the third time point (T40), data from one participant (participant 11) were excluded from analyses involving all three time points. As a result, analyses for the three time points (pre-intervention, postintervention, and one month after the intervention) were conducted on only 10 participants. Analyses involving the first two time points included all 11 participants. The relative abundance of taxonomic and functional groups in stool samples was analyzed, thus enabling observations regarding gut microbiota composition and functionality.

**Figure 1:**
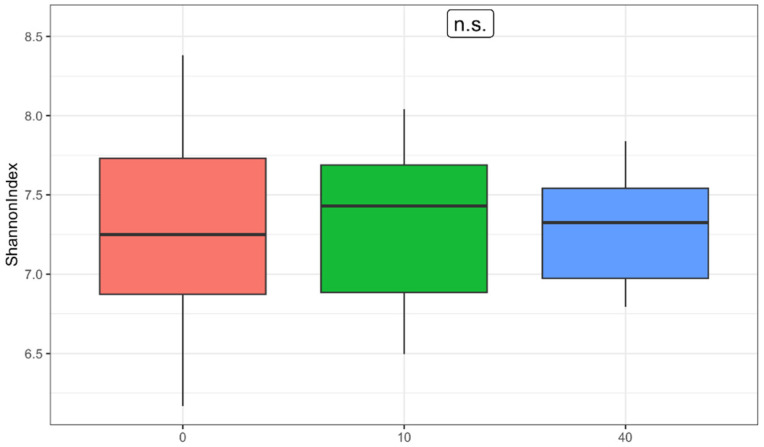
Changes in α-Diversity Across Three Time Points (0 = pre-intervention; 10 = postintervention; 40 = after washout, n.s. = nonsignificant statistical difference).

Shannon’s entropy index was calculated to assess α-diversity. While the median α-diversity increased slightly from 7.23 pre-intervention to 7.38 immediately postintervention, this change was not statistically significant (*p* = 0.832) (Figure 1). After the washout phase, the median returned to 7.32. No statistically significant differences in Shannon index values were observed across the three time points (*p* = 0.895). The range of Shannon index values narrowed after washout, with a pre-intervention range of 6.17–8.38 and a post-washout range of 6.79–7.84. Levene’s test indicated no significant difference in variance between pre- and postintervention (W = 1.62, *p* = 0.225) but a significant difference between pre-intervention and the washout period (W = 4.32, *p* = 0.045).

#### 3.5.1. Gut Microbiota Composition

**Figure 2:**
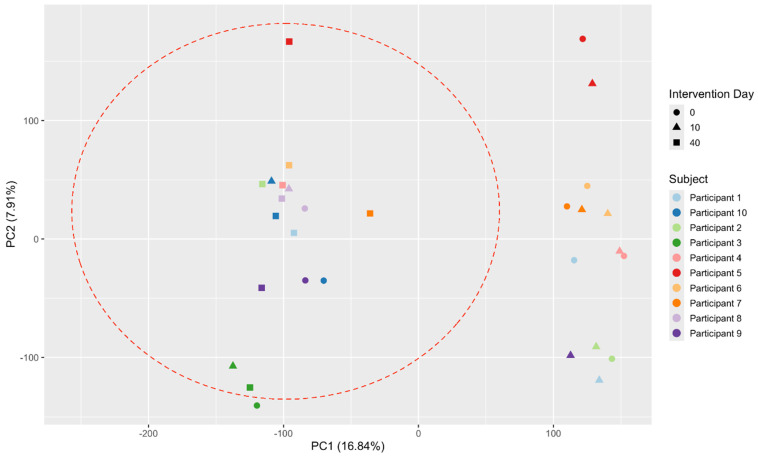
Changes in β-Diversity Across Three Time Points.

The composition of the gut microbiota was analyzed across different time points using β-diversity metrics, specifically Bray–Curtis dissimilarity (Figure 2). Substantial heterogeneity in gut microbiota composition was observed among participants at all time points. Two clusters emerged during the first two time points: one comprising participants 3, 8, 9, and 10 and another comprising participants 1, 2, 4, 5, 6, and 7. However, this heterogeneity decreased at the third time point (after washout), with an increase in similarity across participants (indicated by the red circle) (Figure 2). Despite this trend, no statistically significant differences in β-diversity were detected across time points.

When analyzing the composition of the gut microbiota, statistical analysis revealed significant differences in the relative abundances of bacteria across three time points at all taxonomic levels: from phyla and families to genera. The results concerning phyla and genera are presented in Table 6, ordered by statistical significance. At the phylum level, significant differences were observed in the abundances of five bacterial phyla (*p* < 0.037), including the two main bacterial phyla: Firmicutes and Bacteroidetes. Even when accounting for potential false positives, significant differences remained for four phyla (*q* ≤ 0.045): Firmicutes, Actinobacteria, Lentisphaerae, and Bacteroidetes. When comparing phylum abundances before and one day after the intervention, only the decrease (r = −0.597) in the abundance of the phylum Actinobacteria was significant (*p* = 0.040); however, considering the false-discovery rate (FDR), this is likely a false-positive result (*q* = 0.243). At the family level, significant differences were detected among the three time points in the abundances of 14 bacterial families, with 6 families having highly significant differences (*p* < 0.01). However, unlike at the phylum level, it cannot be excluded that all these differences are false positives (*q* ≥ 0.051). These findings suggest that the intervention may have influenced the composition of the gut microbiota at multiple taxonomic levels, with notable changes in specific phyla and families. However, caution is warranted due to the potential for false-positive results, particularly at the family level.

**Figure 3:**
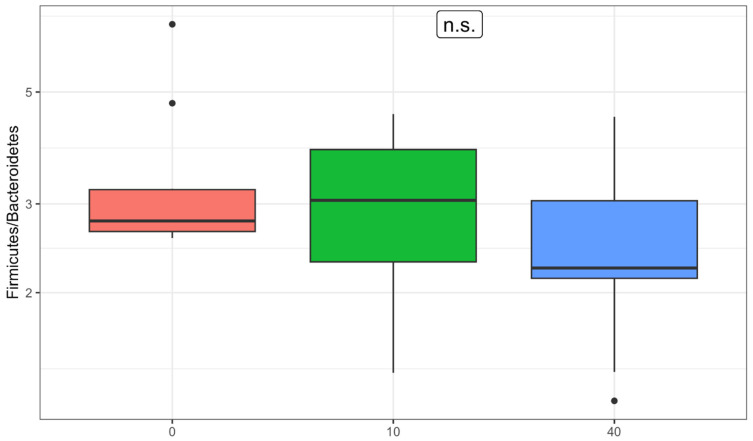
Changes in the Firmicutes/Bacteroidetes (F/B) ratio across the three time points (n.s.: non-significant).

Since significant changes were detected in the abundances of the phyla Firmicutes and Bacteroidetes, changes in the ratio between Firmicutes and Bacteroidetes (commonly referred to as the F/B ratio) were also statistically analyzed (Figure 3). The ratio between these phyla has traditionally been considered a diagnostic marker for obesity, though recent studies [51] have refuted this notion. Today, it is generally regarded as a purely taxonomic indicator of the balance between the two main bacterial groups within the gut microbiota. One day after the intervention, there was an increase in the median F/B ratio, while one month after the intervention, the median F/B ratio decreased to below pre-intervention levels. Differences across the three time points were found to be nearly statistically significant (*p* = 0.057), although FDR correction suggests this is likely a false-positive result (*q* = 0.229). The difference before and one day after the intervention was not statistically significant (*p* = 0.689). For comparison, in a study conducted on the same geographical population (Republic of Croatia) at the same institution and laboratory, all median F/B ratio values across the three time points fell within the interquartile range for this specific geographical population, which is 2.35–4.47.

At the genus level, statistically significant differences across the three time points were detected in as many as 34 genera (*p* < 0.044). However, when accounting for potential false positives using FDR correction, the abundances of only 14 genera appear to be significantly different (*q* ≤ 0.047) (Table 7).

**Figure 4:**
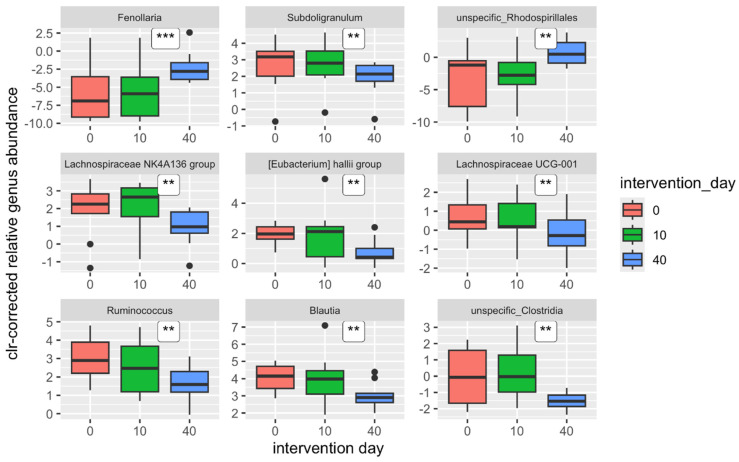
Most significant changes in centered-log-transformed (clr) relative abundances of bacterial genera across the intervention (*** *p* < 0.001; ** *p* < 0.01).

This statistical method analyzed only statistically significant differences among the three time points without considering the direction of change at the genus level. The abundances of the mentioned genera exhibited different behaviors one day and one month after the end of the intervention (Figure 4).

**Figure 5:**
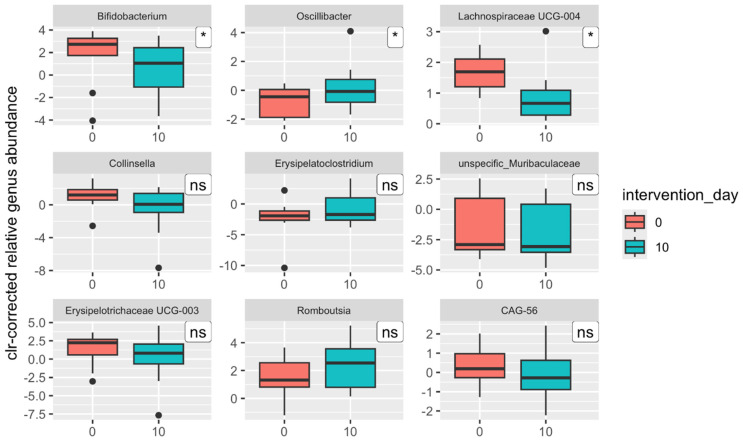
Significant changes in centered-log-transformed (clr) relative abundances of bacterial genera after intervention (* *p* < 0.05; ns: non-significant).

While the abundances of certain genera continuously increased across both time points, such as the genus *Fenollaria* (r = 0.722), the abundances of other genera continuously decreased, as observed with *Subdoligranulum* (r = −0.650), *Ruminococcus* (r = −0.588), and *Blautia* (r = −0.586) (for all three genera, *q* = 0.043). The abundances of some genera, however, decreased one day after the intervention and then increased again, as seen with the nonspecific genus *Rhodospirillales* (r = 0.634), or, conversely, increased first and then decreased, such as *Lachnospiraceae* NK4A136 group (r = −0.627). To better compare with the results of the proof-of-concept study, statistical analyses were performed exclusively on the results before and one day after the intervention at the genus level (Table 8; Figure 5). Significant differences in relative abundances were detected in only three genera one day after the intervention: *Bifidobacterium*, *Oscillibacter*, and *Lachnospiraceae* UCG-004. However, these are likely false-positive results (*q* = 0.958). The decrease in the abundance of the genus *Bifidobacterium* is likely responsible for the decrease in the abundance of the phylum Actinobacteria, to which it belongs, which was also significant one day after the intervention.

#### 3.5.2. Gut Microbiota Functionality

Functional analysis of the gut microbiota was conducted based on the relative abundance of 187 metabolic pathways. Significant differences were detected in 14 pathways across the three time points (7.5% of all pathways), primarily related to nucleotide metabolism (e.g., purine and pyrimidine degradation) (Table 9).

If we examine changes between only two time points, before and one day after the intervention, as done in the proof-of-concept study, the number of significant changes is reduced to just ten metabolic pathways (Table 10). These pathways are involved in fermentation processes, amino acid and carbohydrate degradation, and the synthesis of vitamins B1 and B6. The proportions of amino acid degradation decreased one day after the intervention (r = −0.619 and −0.631), while all other pathways increased (except for the synthesis of thiamine precursor, vitamin B1). One day after the intervention, there were no significant changes in any metabolic pathways associated with nucleotide base metabolism. Furthermore, the FDR correction indicates potential false positives for these detected differences (*q* = 0.708).

To gain better insight into changes in gut microbiota functionality, metabolic pathways were categorized into functional modules using MetaCYC [52] based on class, ontology, and pathway interpretations predicted by PiCRUST2 v2.5.3. Statistical analysis revealed a significant difference across three time points, specifically a reduction, in the proportion of indicators of constipation (*p* = 0.015). However, this association lost significance after false-discovery rate (FDR) correction (Table 11). When examining differences in the proportions of functional modules only before and one day after the intervention, no statistical significance could be detected (lowest *p* = 0.165, *q* = 0.980).

### 3.6. Laboratory Analyses

Laboratory parameter values from venous blood samples were determined at three time points: before the intervention, one day afterward, and one month afterward. Since the distribution of participants’ B12 values did not meet the assumptions of normality according to the Shapiro–Wilk test, the Friedman test was applied to this parameter, whereas repeated-measures ANOVA was used for the remaining four parameters.

Aside from the increase in the leukocyte concentration in serum and the decrease in serum vitamin B12 levels, no consistent changes in other laboratory parameters were observed as a result of the intervention (Table 12). Although the mean leukocyte concentration continued to rise even after the washout phase, the mean B12 concentration returned nearly to its baseline value following washout. Nevertheless, neither the differences in leukocyte concentration (*p* = 0.052) nor those in vitamin B12 (*p* = 0.097) among the three time points reached statistical significance. When analyzing only pre- and postintervention, the increase in leukocyte concentration was nearly significant (*p* = 0.060) and the increase in vitamin B12 concentration significant (*p* = 0.011). Based on the percentages of lymphocytes and neutrophils, it remains unclear which specific leukocyte subgroup contributed to the increased total leukocyte concentration during and after the intervention. Lymphocyte percentage decreased, whereas neutrophil percentage increased immediately after the intervention; however, the latter did not continue to rise following the washout phase.

## 4. Discussion

In this pilot follow-up study, we investigated whether the supplementation of 250 g of sauerkraut over a course of ten days would elicit the same or similar changes in the gut microbiota of active athletes as in an earlier proof-of-concept study [31]. However, the confirmatory study did not yield the same results as the proof-of-concept study.

The original study explored the effects of a 10-day sauerkraut supplementation on gut microbiota composition and functionality in active, professional athletes. The study demonstrated significant and favorable changes in gut microbiota composition, including increased relative abundances of health-promoting anaerobic bacteria, particularly those within the Lachnospiraceae family, which are known short-chain fatty acid (SCFA) producers. These changes were accompanied by alterations in metabolic pathways related to nucleotide metabolism and cell wall synthesis, suggesting improved functionality of the gut microbiota. Interestingly, sauerkraut supplementation did not result in increased α-diversity, contrary to previous studies on fermented foods. Instead, a reduction in the variance of α-diversity values was observed, indicating a stabilizing effect on the gut microbiota. This effect was consistent across participants regardless of baseline microbiota composition, supporting the broad applicability of sauerkraut in gut health interventions. Physiologically, the intervention led to an increase in lymphocyte proportions and a decrease in vitamin B12 levels. Adverse gastrointestinal effects, such as bloating and abdominal pain, were reported during the initial week of supplementation but resolved in all participants by the eighth day. The original study implied that sauerkraut is indeed a synbiotic food, which, when administered in adequate amounts even for a short course of time, can induce a multitude of favorable alterations in the gut microbiota, independent of its baseline composition.

As in the original study, the same confounding factors (diet, sleep, and physical activity) and outcome measures (bowel movement, indigestion, laboratory parameters, and gut microbiota composition and functionality) were assessed. The sole differences in the supplementation protocol and study design between the original and follow-up study were the production year of the sauerkraut, the utilization of novel self-report instruments on dietary intake, and the addition of a washout phase to the study protocol by conducting repeated stool sampling 30 days after sauerkraut supplementation cessation.

Due to the recruitment difficulties experienced during the original study, recreational athletes were included in this follow-up study. The study was conducted on a sample of 11 male active athletes, on average 30 ± 1.56 years old, who were on average athletes with organized training (average athlete classification 2.64 ± 0.98). Although no significant impact of potential confounding factors and similar outcomes were found regarding bowel movement, indigestion, and laboratory parameters, the pilot follow-up study could not confirm the hypotheses stated by proof-of-concept study. Despite all our efforts, we found that despite the identical intervention, sauerkraut could not induce identical favorable changes.

Regarding the gut microbiota composition and functionality, sauerkraut could not be confirmed as a synbiotic fermented food since no uniform significant changes were seen consistently among all of the participants gut microbiota postintervention. Contrary to the original study, no significant changes in β-diversity metrics and no significant increase in the abundances of genera from family Lachnospiraceae and decrease of genera from the family Oscillospiraceae were observed as well as no significant changes in the abundances of metabolic pathways. The identical intervention did not result in even remotely similar results. Furthermore, opposing results were found. In the original study, the median Shannon index decreased postintervention, and in the present study, it increased, albeit in both non-significantly. Genus *Oscillibacter* abundance significantly decreased in the original study and increased in the follow-up study; the same occurred for the relative abundance of genus *Lachnospiraceae* UCG-001. A possible explanation for the discordance of the results could be attributed to the greater heterogeneity of gut microbiota composition before the intervention in the follow-up study when compared to the original study. In the original study, we recruited exclusively professional athletes (average classification 3.9 ± 1.22), which, due to their specific lifestyle, have a distinct gut microbiota profile with less interindividual variability [53,54,55], specifically higher α-diversity and greater abundance of metabolically relevant taxa (family Lachnospiraceae) [56,57,58,59]. Therefore, we observed a greater number of alterations in the same taxonomic and functional groups of the same direction in the original study. This could explain why in the original study, the change in β-diversity was significant, and there was a greater number of significantly altered bacterial genera (eight versus three, *Bifidobacterium*, *Oscillibacter*, and *Lachnospiraceae* UCG-004), metabolic pathways (35 (18.4% of all) versus 10 (5.3% of all)), and functional modules (five versus none).

These results refute one of the key findings of the original study: sauerkraut does not invoke a specific shift in the gut microbiota but rather, like probiotics and other fermented foods, impacts the gut microbiota depending on its baseline composition [60,61].

Curiously, neither the original nor follow-up study found any significant increase in the abundance of lactic acid bacteria (LAB), which is a common phenomenon associated with the intake of fermented food [62]. However, given the microbial population of the sauerkraut sequenced in the first study and the number of LAB demonstrated through cultural methods, this result is not unexpected. Due to the production process, primarily pasteurization, the initial number of LAB is likely lower than in probiotic formulations or fermented products studied in other research, which might have a greater potential to colonize the digestive tract [25,63].

On the other hand, we found in the present study that the relative abundance of the most prominent genera of the sequenced sauerkraut’s microbial population [31] counterintuitively declined postintervention in the host’s gut microbiota, e.g., *Ruminococcus*, *Blautia*, unspecified *Lachnospiraceae*, and *Subdoligranulum*.

However, by including results from a third time point, after a month-long washout period after the intervention, significant changes in gut microbiota composition and functionality were observed, which were greater in magnitude than those seen immediately after the intervention. Although the increase in α-diversity observed immediately postintervention was short-lived, a significant reduction in the range of Shannon index values between samples was noted (*p* = 0.011), from 6.17–8.38 to 6.79–7.84. A similar trend was observed regarding β-diversity: one month postintervention, the gut microbiota composition of participants became more similar than in the pre-intervention measurement. When comparing the relative abundance of taxonomic groups across three time points rather than just pre- and postintervention, the number of significant changes increased substantially. Four phyla—Firmicutes, Actinobacteria, Lentisphaerae, and Bacteroidetes (*q* ≤ 0.045)—showed significant differences in contrast to only one phylum (Actinobacteria) postintervention (*q* = 0.243). In addition, ten bacterial genera (*q* ≤ 0.047) demonstrated significant changes compared with just three (*q* = 0.958) postintervention. The same applied to functional groups: significant differences were detected in 14 metabolic pathways (7.5% of all pathways), most of which were associated with nucleotide metabolism, without indications of false positivity (*q* ≤ 0.481). These findings underscore the intervention’s substantial impact following the washout period.

These results indicate that supplementation with sauerkraut in this sample of recreational athletes had certain significant long-term effects on the composition and functionality of their gut microbiota, regardless of its initial state. In the pilot follow-up study, we could not replicate the short-term synbiotic effects of sauerkraut seen in the original study; however, its long-term effects on both composition as well as functionality were notable. We can state that the supplementation of 250 g of sauerkraut over a course of 10 days can induce significant alterations in the gut microbiota that are detectable by sequencing methods even one month after cessation of sauerkraut intake. Our research suggests that the synbiotic effect of sauerkraut is not transient, lasting at least for a whole month postintervention.

Contrary to its short-term effects on the gut microbiota, the pilot follow-up study confirmed the short-term effects of sauerkraut on digestion and indigestion, as seen in the original study. The probability of normal stool (Bristol stool types 3 and 4) increased after one week, although this finding was not statistically significant, as in the original study. The highest incidence of reported adverse effects in the follow-up study occurred on days 5 and 7 of the intervention, while in the original study, it occurred on days 5 and 6. When calculated relative to all participant-days for the respective study (proof-of-concept = 100 days; pilot follow-up = 110 days), our findings indicate that sauerkraut supplementation at the specified dose of 250 g carries the highest probability of causing bloating (approximately 16%), with much lower probabilities of diarrhea (3.3%) or abdominal pain (2.3%). Our results suggest that while adverse effects such as bloating are relatively common during the early stages of sauerkraut supplementation, they diminish substantially over time, with minimal adverse effects observed on days 8 to 10.

The fact that participants in the present study also reported constipation and nausea as adverse effects, unlike in the original study, could also be attributed to the greater heterogeneity of their gut microbiota when compared to participants of the original study.

It seems that with the regular intake of sauerkraut, the digestion tract and the gut microbiota apparently adapt to its compounds, and then, sauerkraut becomes safe to use, similarly to probiotics and other fermented food. Hence, we can confirm the recommendation for a seven-day long adaptation period for sauerkraut supplementation from the original study, which is the also case for probiotics [64,65,66].

Regarding laboratory parameters, a nearly significant increase in leukocyte concentration and a significant decrease in serum vitamin B12 levels concentration was observed postintervention in the present study. One month after the intervention, serum vitamin B12 levels normalized (*p* = 0.097), while leukocyte concentration showed an almost significant further increase (*p* = 0.052). The significant decrease in vitamin B12 levels is, as seen in the original study (*p* = 0.012), probably is a short-term effect of sauerkraut supplementation, which warrants further research.

There are several substantial limitations of this study. The main limitation of the pilot follow-up study was that it was repeated with a very small subject number. This is represented by high false-positive rates regarding the many findings of the study. Studies on greater samples with different population characteristics (age, gender, and health status) are required to yield a clearer picture of the synbiotic effect of sauerkraut.

Due to knowledge, population, and practical gaps, we cannot conclude whether the observed long-term effects of sauerkraut supplementation on the abundance of taxonomic and functional groups are favorable.

Although the complete amplicon sequencing process utilized the same sampling, sequencing, and bioinformatical methods, there is the possibility of technological bias due to non-foreseeable factors around this very delicate diagnostic method. Since the original and follow-up studies were performed at different times, there is the slight chance that minor technical issues during sampling, the handling of the samples, or sequencing caused the discrepancy between the results. One other important limitation of the pilot follow-up study is the fact that the investigated sauerkraut was pasteurized. This could also explain why no increase in the relative abundances of lactic acid bacteria was observed and why the abundance of certain genera, which are abundant in the sauerkraut microbial population, did not increase in the host’s gut microbiota after the intervention.

Additionally, one should be cautious when calling the used sauerkraut a synbiotic since it rather contained pre- and postbiotics and not probiotics.

## 5. Conclusions

The pilot follow-up study could not reciprocate the findings on sauerkraut’s short-term effects on the gut microbiota from the original study. Our results jointly do confirm that the short-term effects depend on the baseline status of the gut microbiota but suggest that in homogenous populations, such as professional athletes, the size and direction of effect is more uniform, depending on α-diversity and the abundance of specific taxonomic (Lachnospiraceae and Oscillospiraceae) and functional groups. The follow-up study discovered that sauerkraut supplementation can be associated with long-term effects on the gut microbiota composition and functionality, which are persistent after supplementation is terminated. We confirmed with the pilot follow-up study that sauerkraut supplementation requires a minimal seven-day adaptation period, during which indigestion can occur. Sauerkraut supplementation can induce a short-term decrease in serum vitamin B12 levels. Further studies with greater sample sizes and smaller sauerkraut daily doses to minimize adverse effects are warranted to deliver more evidence for the use of sauerkraut in sports nutrition for gut microbiota personalization. It is essential to see which athletes’ gut microbiota respond or do not respond to sauerkraut supplementation in both the short and long term and whether the same effects could be obtained with only sauerkraut brine as the liquid component.

## Figures and Tables

**Table 1:** Participants.

Participant	Sex	Age (Years)	Sport	Years in Sport	Athlete Classification
1	M	32	Strength training	22	Level 2
2	M	31	Strength training	18	Level 2
3	M	30	Football	23	Level 2
4	M	27	Football	24	Level 5
5	M	32	Rugby	25	Level 4
6	M	30	Strength training	19	Level 2
7	M	30	Strength training	16	Level 2
8	M	30	Athletics	20	Level 3
9	M	29	Football	18	Level 3
10	M	28	Strength training	14	Level 2
11	M	32	Hiking	20	Level 2
Mean, SD		30 ± 1.56		20.8 ± 5.69	2.64 ± 0.98

SD: standard deviation.

**Table 2:** Physical Activity and Sleep.

Confounding Factor	Before Intervention	During Intervention	Difference (*p*-Value)
Training frequency (sessions per week)	4.18 ± 1.72	4.0 ± 2.02	0.472
Training duration (minutes/day)	64.09 ± 20.35	62.01 ± 16.2	0.559
Sleep duration (hours)	7.78 ± 0.38	7.91 ± 0.37	0.263

**Table 3:** Dietary Intake.

	Before Intervention (Mean ± SD)	During Intervention (Mean ± SD)	Difference (*p*-Value)
Energy intake (kcal)	2918.50 ± 171.64	2965.42 ± 227.94	0.469
Protein intake (g)	153.88 ± 16.58	157.82 ± 13.58	0.472
Protein intake (g/kg)	1.68 ± 0.24	1.73 ± 0.21	0.631
Carbohydrate intake (g)	324.32 ± 31.28	318.69 ± 39.90	0.378
Carbohydrate intake (g/kg)	3.57 ± 0.32	3.56 ± 0.32	0.785
Fat intake (g)	98.02 ± 12.59	103.78 ± 10.64	0.092
Fat intake (% energy)	31.52 ± 2.32	31.63 ± 1.81	0.914
Fiber intake (g)	25.25 ± 2.15	29.04 ± 3.06	0.011 *
Fiber intake (g/1000 kcal)	8.88 ± 0.66	10.21 ± 1.82	0.020 *

* indicating *p* < 0.05, g: grams; kg: kilograms; kcal: kilocalories.

**Table 4:** Questionnaire Results.

Questionnaire	Before Intervention	During Intervention	Difference (*p*-Value)
ADI	60.09 ± 14.77	58.73 ± 12.37	1.000
MEDAS	6.09 ± 2.43	6.64 ± 2.11	0.104
SQM	7 ± 1.79	7.45 ± 2.58	0.073

**Table 5:** Bowel movements and adverse effects during intervention.

Participants (N) Indicating BST							Probability for BST 3 and 4	Participants (N) Indicating Adverse Effects				
Day	1	2	3	4	5	6	HR, CI, *p*-Value	Bloating	Diarrhea	Pain	Constipation	Nausea
1	0	4	2	3	2	0	0.45, [16.0%, 74.9%], 1.000	1	0	0	0	0
2	0	3	3	3	2	0	0.55, [25.1%, 84.0%], 1.000	2	0	0	0	1
3	0	2	3	3	2	1	0.55, [25.1%, 84.0%], 1.000	3	0	0	0	0
4	1	1	2	5	1	1	0.64, [35.2%, 92.1%], 0.549	1	0	0	0	1
5	0	1	2	3	3	2	0.45, [16.0%, 74.9%], 1.000	3	2	0	1	1
6	0	1	3	3	2	2	0.55, [25.1%, 84.0%], 1.000	2	0	0	0	0
7	0	1	2	3	3	2	0.45, [16.0%, 74.9%], 1.000	3	1	0	0	0
8	0	2	3	4	2	0	0.64, [35.2%, 92.1%], 0.549	0	0	1	0	0
9	0	1	3	5	2	0	0.73, [46.4%, 99.0%], 0.227	1	0	0	0	0
10	0	2	3	5	1	0	0.73, [46.4%, 99.0%], 0.227	1	0	1	0	0

HR: hazard ratio; CI: 95% confidence interval; BST: Bristol stool type.

**Table 6:** Changes in the abundances of phyla and families due to the intervention.

Phylum	*p*-Value	Correlation Coefficient	FDR
Firmicutes	0.004	−0.600	0.028
Actinobacteria	0.016	−0.521	0.045
Lentisphaerae	0.025	0.489	0.045
Bacteroidetes	0.026	−0.485	0.045
Cyanobacteria	0.037	0.457	0.052
Proteobacteria	0.175	−0.307	0.205
Desulfobacterota	0.687	−0.093	0.687
Family			
Unspecified Rhodospirillales	0.002	0.647	0.051
Ruminococcaceae	0.003	−0.609	0.051
Streptococcaceae	0.004	−0.603	0.051
Unspecified Clostridia	0.006	−0.576	0.056
Peptostreptococcaceae	0.007	−0.567	0.056
Butyricicoccaceae	0.009	−0.558	0.056
Unspecified Alphaproteobacteria	0.010	0.548	0.056
Unspecified Gastranaerophilales	0.011	0.541	0.056
Lachnospiraceae	0.013	−0.534	0.056
[Eubacterium] coprostanoligenes group	0.017	−0.514	0.063
Family XI Tissierellales	0.017	0.513	0.063
Victivallaceae	0.022	0.497	0.073
Clostridiaceae	0.028	−0.478	0.087
Anaerovoracaceae	0.035	−0.461	0.101
Erysipelatoclostridiaceae	0.054	−0.427	0.144
Veillonellaceae	0.081	−0.390	0.202
Muribaculaceae	0.095	−0.374	0.223
Eggerthellaceae	0.147	−0.328	0.321
Oscillospiraceae	0.153	−0.323	0.321
Barnesiellaceae	0.163	0.316	0.326
Christensenellaceae	0.200	−0.291	0.381
Unspecified RF39	0.222	0.278	0.404
Acidaminococcaceae	0.253	0.261	0.440
Bacteroidaceae	0.268	−0.253	0.446
Enterobacteriaceae	0.279	−0.248	0.446
Sutterellaceae	0.321	−0.228	0.494
Marinifilaceae	0.386	0.200	0.562
Bifidobacteriaceae	0.397	−0.195	0.562
Erysipelotrichaceae	0.407	−0.191	0.562
Prevotellaceae	0.502	0.155	0.670
Unspecified Clostridia vadinBB60 group	0.539	0.142	0.695
Desulfovibrionaceae	0.582	−0.128	0.727
Monoglobaceae	0.684	−0.095	0.809
Unspecified Clostridia UCG-014	0.688	0.093	0.809
Lactobacillaceae	0.739	−0.077	0.826
Tannerellaceae	0.744	−0.076	0.826
Coriobacteriaceae	0.871	−0.038	0.919
Pasteurellaceae	0.873	−0.037	0.919
Rikenellaceae	0.944	0.016	0.968
Selenomonadaceae	0.990	0.003	0.990

FDR: false-discovery rate.

**Table 7:** Most significant changes at the genus level due to the intervention.

Genus	*p*-Value	Correlation Coefficient	FDR
*Fenollaria*	<0.001	0.722	0.019
*Subdoligranulum*	0.001	−0.650	0.043
Unspecified *Rhodospirillales*	0.002	0.634	0.043
*Lachnospiraceae* NK4A136 group	0.002	−0.627	0.043
*[Eubacterium] hallii* group	0.003	−0.619	0.043
*Lachnospiraceae* UCG-001	0.005	−0.591	0.043
*Ruminococcus*	0.005	−0.588	0.043
*Blautia*	0.005	−0.586	0.043
Unspecified *Clostridia*	0.006	−0.583	0.043
*Romboutsia*	0.006	−0.581	0.043
*Peptoniphilus*	0.006	0.580	0.043
*Streptococcus*	0.006	−0.576	0.043
*Butyricicoccus*	0.006	−0.575	0.043
Unspecified *Lachnospiraceae*	0.008	−0.564	0.047

FDR: false-discovery rate.

**Table 8:** Significant changes in centered-log-transformed (clr) relative abundances of bacterial genera after intervention.

Genus	*p*-Value	Correlation Coefficient	FDR
*Bifidobacterium*	0.028	−0.630	0.958
*Oscillibacter*	0.030	0.625	0.958
*Lachnospiraceae* UCG-004	0.047	−0.582	0.958

FDR: false-discovery rate.

**Table 9:** Significant Changes in Metabolic Pathways Across Three Time Points.

Metabolic Pathway	*p*-Value	Correlation Coefficient	FDR
Degradation of purine nucleobases I (anaerobic)	0.005	−0.583	0.424
Superpathway of purine deoxyribonucleoside degradation	0.006	−0.577	0.424
Methanogenesis from acetate	0.007	−0.570	0.424
Acetylene degradation	0.010	−0.549	0.424
CMP-3-deoxy-D-manno-octulosonate biosynthesis I	0.011	0.541	0.424
Superpathway of aspartate	0.019	−0.507	0.481
Superpathway of thiamin diphosphate biosynthesis I	0.025	0.489	0.481
Lipid IVA biosynthesis	0.026	0.484	0.481
Queuosine biosynthesis	0.029	0.476	0.481
Superpathway of O-antigen building blocks biosynthesis from GDP-mannose	0.029	0.475	0.481
Saturated fatty acid elongation	0.033	0.468	0.481
Superpathway of pyrimidine deoxyribonucleoside degradation	0.034	−0.465	0.481
Sucrose degradation III (sucrose invertase)	0.034	−0.464	0.481
Guanosine nucleotide degradation III	0.036	−0.459	0.481
Superpathway of *N*-acetylglucosamine, *N*-acetylmannosamine, and *N*-acetylneuraminate degradation	0.039	−0.454	0.481

FDR: false-discovery rate.

**Table 10:** Significant alterations of metabolic pathways the day after intervention.

Metabolic Pathway	*p*-Value	Correlation Coefficient	FDR
Pyruvate fermentation to propanoate I	0.005	0.752	0.708
L-histidine degradation I	0.022	0.651	0.708
S-adenosyl-L-methionine cycle I	0.028	−0.631	0.708
Glycolysis I (from glucose 6-phosphate)	0.028	0.631	0.708
Homolactic fermentation	0.031	0.622	0.708
Glycolysis II (from fructose 6-phosphate)	0.031	0.621	0.708
Superpathway of L-alanine biosynthesis	0.032	−0.619	0.708
Mixed acid fermentation	0.040	0.598	0.708
Thiazole biosynthesis I (*E. coli*)	0.045	−0.587	0.708
Superpathway of pyridoxal 5′-phosphate biosynthesis and salvage	0.046	0.585	0.708

FDR: false-discovery rate.

**Table 11:** Alterations in modules of gut microbiota functionality.

Functional Module	*p*-Value	Correlation Coefficient	FDR
“Indicators of Constipation”	0.015 *	−0.525	0.218
“Indicators of Inflammation”	0.166	0.314	0.632
“Fat Breakdown”	0.168	−0.313	0.632
“Carbohydrate Breakdown [Sugars]”	0.187	−0.299	0.632
“Intestinal Barrier Function”	0.284	0.245	0.632
“Energy Metabolism and Hyperacidity”	0.292	−0.241	0.632
“Appetite and Cholesterol Levels”	0.307	−0.234	0.632
“Lactose Intolerance”	0.337	−0.220	0.632
“Vitamin K Production”	0.426	−0.184	0.710
“Cytotoxins”	0.509	0.152	0.755
“Protein Fermentation”	0.553	−0.137	0.755
“Carbohydrate Breakdown [Polysaccharides]”	0.694	0.091	0.867
“Vitamin B12 Production”	0.831	0.049	0.955
“Fructose Intolerance”	0.891	−0.032	0.955
“Sleep and Mental State”	0.970	0.009	0.970

* *p* < 0.05; FDR: false-discovery rate.

**Table 12:** Laboratory Parameters.

Parameter	Unit	Pre-Intervention (Mean ± SD)	Postintervention (Mean ± SD)	After Washout (Mean ± SD)	*p*-Value
Leukocytes	(10^9^/L)	5.35 ± 1.16	5.9 ± 1.05	6.39 ± 1.7	0.052
Lymphocytes	(%)	40.19 ± 4.89	36.28 ± 6.4	36.4 ± 8.46	0.228
Neutrophils	(%)	47.65 ± 4.72	51.92 ± 6.17	51.36 ± 7.08	0.196
Vitamin B12	(mg/mL)	366.1 ± 57.31	326.3 ± 51.18	355.7 ± 56.02	0.097
Folic acid	(mg/mL)	32.24 ± 7.74	32.94 ± 13.14	33.43 ± 8.91	0.913

SD: standard deviation.

## Data Availability

The original contributions presented in this study are included in the article. Further inquiries can be directed to the corresponding author.

## Abbreviations

The following abbreviations are used in this manuscript:

MDPI	Multidisciplinary Digital Publishing Institute
DOAJ	Directory of Open Access Journals
TLA	Three-letter acronym
LD	Linear dichroism
FDR	False-discovery rate
MEDAS	Mediterranean Diet Adherence Scanner
ADI	Athlete diet index
SQM	Short Questionnaire on Mediterranean Diet Adherence and Diet Sustainability
